# The Cranial Neural Crest in a Multiomics Era

**DOI:** 10.3389/fphys.2021.634440

**Published:** 2021-03-01

**Authors:** Vanessa Chong-Morrison, Tatjana Sauka-Spengler

**Affiliations:** Radcliffe Department of Medicine, Weatherall Institute of Molecular Medicine, University of Oxford, Oxford, United Kingdom

**Keywords:** neural crest, multiomics, gene regulatory network, non-coding, interactome, transcriptome, epigenome

## Abstract

Neural crest ontogeny plays a prominent role in craniofacial development. In this Perspective article, we discuss recent advances to the understanding of mechanisms underlying the cranial neural crest gene regulatory network (cNC-GRN) stemming from *omics*-based studies. We briefly summarize how parallel considerations of transcriptome, interactome, and epigenome data significantly elaborated the roles of key players derived from pre-*omics* era studies. Furthermore, the growing cohort of cNC multiomics data revealed contribution of the non-coding genomic landscape. As technological improvements are constantly being developed, we reflect on key questions we are poised to address by taking advantage of the unique perspective a multiomics approach has to offer.

## 1. Introduction

Gene regulatory networks (GRNs) coordinate the expression of genes encoding transcription factors (TFs), cell signaling pathway components and differentiation effectors in genetic cascades mediated by *cis*-regulatory elements (Levine and Davidson, 2005). GRNs present a unique perspective in the understanding of developmental pathways and mechanisms by focusing on the regulated activity of genes within a defined cellular context. The lengthy process of neural crest (NC) development, that starts at the end of gastrulation and proceeds into late organogenesis has been proposed to be orchestrated by a multi-module GRN (Sauka-Spengler and Bronner-Fraser, 2008; Simões-Costa and Bronner, 2015). Broadly-speaking, Wnt, Fgf, and Bmp signals at the neural plate border activate expression of genes from the *Msx, Pax*, and *Zic* families during NC induction (Ikeya et al., 1997; LaBonne and Bronner-Fraser, 1998; Monsoro-Burq et al., 2003, 2005; Lewis et al., 2004; Schumacher et al., 2011). Pax3 and Zic1 activate expression of *bona fide* NC factors, such as *Snai1* and *FoxD3*, thus driving the onset of NC specification defined by the expression of *Tfap2, Id, Myc, Myb, SoxE*, and *Ets* gene family members (Luo et al., 2003; Sato et al., 2005; Hong and Saint-Jeannet, 2007; Sauka-Spengler et al., 2007; Milet et al., 2013; Schock and LaBonne, 2020). The persisting expression of these TFs, as well as the downstream activation of cadherins, integrins, signaling receptors and metalloproteases, subsequently lead to epithelial-to-mesenchymal transition (EMT) and delamination of NC cells from the dorsal neural tube. Cranial NC (cNC) cells migrate via canonical, well-established pathways to their final destinations within the vertebrate embryo's head. Unlike the cNC that can give rise to ectomesenchymal derivatives (forming the cartilage, bones, and connective tissues of the craniofacial skeleton), non-cranial, more posterior NC (vagal, trunk, sacral) form mostly neuronal derivatives such as the sensory neurons and glia in the dorsal root ganglia, sympathetic ganglia and enteric nervous system. Although cranial and trunk NC express similar groups of early marker genes, some distinct TFs (e.g., *Sox8, Tfap2*β, *Ets1*) driving cranial vs. trunk NC identity have been described (Simoes-Costa and Bronner, 2016). Recent work suggested that elaborate NC-GRN was progressively established during the evolution of vertebrates with trunk-like circuits being in place first (Martik et al., 2019). However, detailed analysis and understanding of the trunk NC-GRN remains to be done.

The dawn of the -*omics* era has contributed significantly to the elaboration and refinement of existing GRNs. Coupled with an increasing catalog of sequenced genomes, genome-wide approaches heralded an explosion of exploratory studies that not only recapitulated previous knowledge but also increased the rate of identification of novel developmental players. In the avian NC, RNA-seq identified multiple genes not known previously to be expressed in the migratory cNC such as *Lmo4, RxrG, Ltk*, and *Col9a3* (Simões-Costa et al., 2014). Furthermore, work to compare the transcriptomes of trunk and cNC populations coupled with subsequent functional assays led to identification of a cranial-specific module in the migratory cNC consisting of Brn3c, Lhx5, Dmbx1 in the neural plate border; Sox8, Tfap2β in premigratory NC; and Ets1 (activated by Tfap2β) (Simoes-Costa and Bronner, 2016). Overexpression of these factors in the trunk NC resulted in reprogramming of their identity, highlighted by the ectopic activity of a cNC-specific enhancer SOX10E2 (Betancur et al., 2010) and increased expression of chondrocyte-related genes *Runx2* and *Alx1*. Importantly, gene modules are not limited to the “gross” distinction between trunk vs. cNC, as key differences in the molecular signature between cNC cells from different axial-levels could also be detected using RNA-seq (Lumb et al., 2017). Altogether, these studies exemplify the amenability of the cNC-GRN to be interrogated via an -*omics*-type approach for the desired outcome of identifying gene modules specific to subpopulations within the cNC.

Such efforts to resolve spatiotemporal dynamics of the cNC-GRN were further strengthened by emerging single cell technologies. Single cell RNA-seq (scRNA-seq) of 406 cNC cells isolated from the avian embryo identified a fraction of invasive front cells (Trailblazers) with a distinct molecular signature that persisted through migration, therefore bringing to light subpopulations within one cell type with seemingly similar cell behavior (Morrison et al., 2017). Strikingly, scRNA-seq analysis of 1345 murine cNC cells detected a subtle but observable discrete cell state where cNC cells displayed a bias toward neuronal vs. mesenchymal fate during delamination from the neural tube (Soldatov et al., 2019). This finding refined and elaborated the long-held model of sequential (induction, specification, delamination, differentiation) developmental events underlying the cNC-GRN and demonstrated the fluid nature of cNC ontogeny at the transcriptional level. Pertinently, scRNA-seq addressed a constantly debated question within the field concerning hetero- or homogeneity of premigratory NC cells. Investigation of transcriptional heterogeneity of premigratory NC cells *in vivo* using scRNA-seq of FAC-sorted *foxd3*-positive cNC cells from 5 to 6 ss zebrafish embryos (Lukoseviciute et al., 2018) failed to identify multiple specific NC subpopulations but singled-out a small cluster of NC cells which expressed low levels of factors key to NC specification—*zic2b, tfap2a, sox10, twist1b, ets1, pax3a*, including *foxd3*. These cells expressed high levels of stem-cell state (“stemness”) factors such as *snai1a, vent, vox*, and *cx43.4*, suggesting that they may represent non-specified cNC progenitors maintained in premigratory cNC. This finding echoes an observation made by machine learning-based image analysis that clustered cNC cells based on expression of a selected panel of genes (including pluripotency and NC markers) within similar-staged avian embryos (Lignell et al., 2017).

From a GRN perspective, scRNA-seq called into question the existence of one unifying NC-GRN or multiple NC-GRNs working in concert with each other to drive NC development. Previous iterations of the NC-GRN were largely based on candidate gene approach studies, thus representing a summation of parts averaged across the NC as a whole. ScRNA-seq dissected this “unified” NC-GRN model into their parts, by revealing subpopulations with distinct molecular signatures (even if they were pre-enriched for cNC) hinting at “multiple” NC-GRNs. In particular, comprehensive analysis of NC enhancer modules in the cranial region suggested that NC gene regulatory circuits controlling neuronal derivatives are established much earlier in the embryo and use non-exclusive *cis*-regulatory elements shared with neural programmes. In contrast, regulatory circuits underlying mesenchymal/canonical NC gene expression are laid down later when neural tissue is already defined. These later circuits use an intermediary cohort of enhancers active exclusively in the NC (Williams et al., 2019). Such dichotomy in regulatory element modules and NC circuits was also uncovered in the vagal NC giving rise to the enteric nervous system. The neuronal derivative programme was pleiotropic, whereas the GRN underlying neural/glial/mesenchymal derivatives was newly established and utilized by NC cells only (Ling and Sauka-Spengler, 2019).

Methodologically, “first-generation” scRNA-seq studies prior to Williams et al. (2019) utilized FAC-sorting followed by sequencing of full-length mRNA transcripts on a relatively small number of single cells, an approach that although robust, was also laborious and limited in statistical power for sensitive clustering of subpopulations of cells. Nonetheless, they played an important role in priming the NC field for droplet-based technologies allowing a significantly higher number of cells (by the thousands, not hundreds) to be profiled at any one time, therefore bypassing this limitation. The powerful use of the latter approach was also demonstrated in the proto-vertebrate *Ciona intestinalis*, where the resolution achieved enabled identification of an ancestral *Six, Msx*, and *Pax* regulatory module shared between cranial placodes and NC in vertebrates (Horie et al., 2018).

## 2. *cis*-Regulatory Elements Unify NC Gene Modules

Positive *cis*-regulatory elements, also known as enhancers, serve as important “switches” within GRN modules by integrating inputs/binding of upstream factors in order to coordinate output/expression of downstream targets. SOX10E1 and SOX10E2 enhancers, situated 1 kb downstream of the coding region for NC master regulator *Sox10* (Kelsh, 2006; Sauka-Spengler and Bronner-Fraser, 2008; Schock and LaBonne, 2020) have been shown to control the expression of *Sox10* in the chicken embryo (Betancur et al., 2010). Both enhancers demonstrated distinct spatiotemporal activity, where SOX10E2 alone was active in early delaminating cNC cells. Mutations at key binding motifs identified in SOX10E2, knockdown of upstream TFs and chromatin immunoprecipitation (ChIP) experiments confirmed Sox9, Ets1, and cMyb proteins as transcriptional inputs for endogenous *Sox10* gene expression. Similarly, two enhancers, NC1 and NC2, located 20 and 44 kb upstream of the NC specifier *FoxD3* have been shown to control *FoxD3* gene expression in the avian embryo (Simões-Costa et al., 2012). NC1 (but not NC2) was found to be active in the premigratory cNC, but its activity diminished during migration and no activity could be detected caudal to rhombomere 3. Knockdown of upstream factors such as *Pax7, Msx1, Ets1*, and *Zic1* confirmed their participation in the *FoxD3* module underlying gene regulation between trunk and cNC—Ets1 demonstrated cranial-specific control of NC1, Zic1 controlled vagal- and trunk-specific activity of NC2, while Pax7 and Msx1 inputs where shared between NC1 and NC2. Altogether, these case studies presented clear evidence for the role of enhancers in maintaining spatiotemporal expression of developmentally-regulated cNC genes. They spearheaded higher throughput genome-wide characterization of the global cNC landscape using approaches such as ChIP-seq (Barski et al., 2007) and ATAC-seq (Buenrostro et al., 2013) to profile large cohorts of NC enhancers both in the embryo and *in vitro*.

It has been shown that developmental enhancers display specific histone signatures, such as H3K27ac and H3K4me1, indicative of their active vs. poised chromatin states (Creyghton et al., 2010). Furthermore, several studies have demonstrated that chromatin remodelers and their associated histone marks are regulated during NC development (reviewed by Strobl-Mazzulla et al., 2012). Large-scale epigenomic mapping using p300, H3K27Ac, H3K4me1, and H3K4me3 enrichment profiles successfully facilitated the identification of enhancer elements in human cNC cell culture, uncovering the association of a key NC specifier, TFAP2A, with permissive chromatin landscape at putative NC enhancers (Rada-Iglesias et al., 2012). This coupling between epigenetic modulation of enhancers and function was further strengthened by studies in mouse embryonic stem cells (mESC) elucidating the mechanism by which another NC specifier, FOXD3, acted to decommission enhancers via recruitment of specific chromatin remodelers (Krishnakumar et al., 2016; Respuela et al., 2016). *In vivo*, epigenome profiling of subpopulations of mouse cNC cells exposed the differences in chromatin signature reflective of their positional identity (Minoux et al., 2017).

## 3. Multiomics and Rebuilding the cNC-GRN

**Figure 1:**
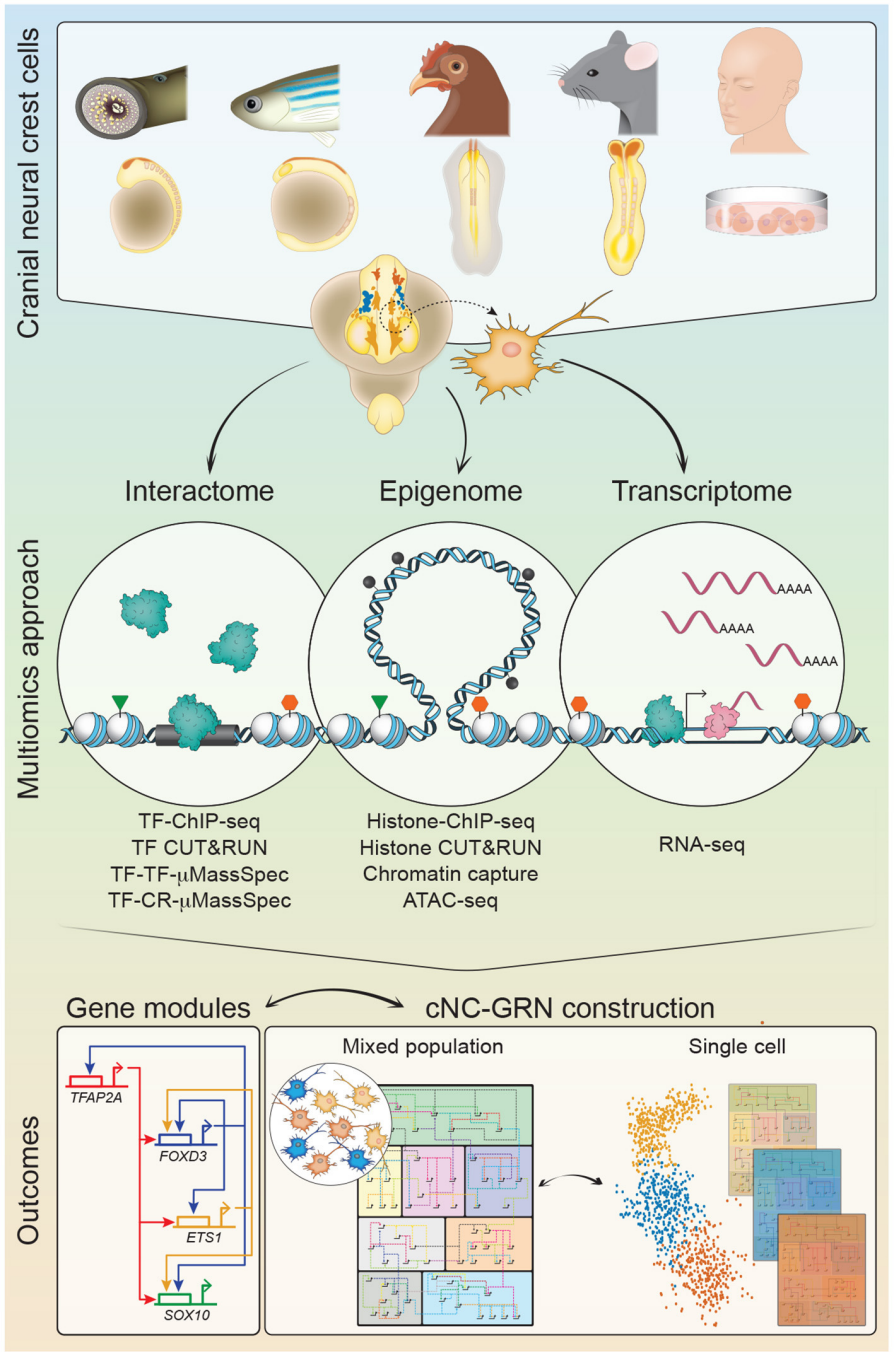
A multiomics approach for construction of the cranial neural crest gene regulatory network (cNC-GRN). CNCCs from *in vivo* non-human embryo models and human pluripotent stem cell differentiation *in vitro* model were subjected to multiomics interrogation for global-level information. Interactome analyses resolve TF interactions to the genome (TF-ChIP-seq, TF CUT&RUN), other TFs (TF-TF-μMassSpec), or CRs (TF-CR-μMassSpec). Epigenome analyses reveal enhancers and promoters defined by regions of accessible chromatin (ATAC-seq) and/or specific histone modifications (Histone-ChIP-seq, Histone CUT&RUN). CUT&RUN is an alternative method to ChIP-seq that has its utility demonstrated in the chick embryo NC (Skene and Henikoff, 2017; Rothstein and Simoes-Costa, 2020). Direct epigenomic relationships between promoters and enhancers are obtained by profiling their physical proximity (Chromatin capture). Transcriptome analysis provides snapshot of expressed genes. Parsing of all the datasets results in substantial number of gene modules to elaborate on the cNC-GRN, especially if coupled with single cell technologies for subpopulation resolution. CNCC, cranial neural crest cell; TF, transcription factor; CR, chromatin remodeler; μMassSpec, micro mass spectrophotometry.

The cNC-GRN has benefited from the substantial body of *in vivo* data, occasionally complemented by *in vitro* studies, from numerous labs over the past few decades. Pre-*omics*, a glaring knowledge gap persisted as experimental limitations lacked detail on the extent of the inter-connectivity between gene modules. Taking full advantage of the multiomics revolution and the demonstration of its utility in proof-of-principle characterization of early human embryos (Li et al., 2018), a multiomics approach was employed in multiple model organisms to re-examine the cNC-GRN. These studies sought to parse substantial biological information obtained from multiple levels: the NC genome (regions of open chromatin), transcriptome (RNA transcripts, including nascent transcripts), epigenome (chromatin modifications, chromatin-looping), and interactome (protein-DNA or protein-protein interactions) into workable hypotheses to test novel mechanisms, gene modules and players (Figure 1). For instance, *omics* interrogation of chromatin accessibility and looping during cNC development, in combination with transcriptional dynamics analyzed at both population and single-cell level in chick revealed a rich tapestry of gene modules. This not only provided insight into subcircuits underlying cNC heterogeneity (with identification of some novel inputs) but also enabled reverse engineering of gene regulatory circuits for every gene expressed, thus facilitating reconstruction of the global NC-GRN with unrivaled resolution (Williams et al., 2019). Combined with gold standard molecular techniques in the embryo such as enhancer screens and knockout experiments (Hockman et al., 2019; Ling and Sauka-Spengler, 2019; Williams et al., 2019), the collective result yielded as powerful resources with the potential to not only recapitulate previous work but also significantly expand on them. Ultimately, these studies accelerate progress for the myriad of biological questions-of-interest within the NC research community with far-reaching implications in biology, evolution, health and disease. Here, we briefly highlight recent findings in cNC-GRN biology resulting from multiomics.

### 3.1. Molecular Mechanism of cNC Pioneer Factors

FOXD3 transcription factor is an important player in the NC-GRN (Lister et al., 2006; Montero-Balaguer et al., 2006; Stewart et al., 2006; Wang et al., 2011c) with evidence in embryonic stem cells documenting its possible cellular function as both a repressor and activator (Pohl and Knöchel, 2001; Yaklichkin et al., 2007; Krishnakumar et al., 2016; Respuela et al., 2016). A transgenic zebrafish line where the *foxd3* locus has been disrupted with a Citrine or mCherry fluorophore (Hochgreb-Hägele and Bronner, 2013) was used to characterize FoxD3 bimodal properties within its native context in a developing embryo. By performing genetic crosses between *foxd3*-mCherry and *foxd3*-Citrine heterozygote parents, *foxd3*-Citrine heterozygote and *foxd3*-mCherry/Citrine homozygote knockout NC cells were isolated by FACS for downstream multiomics analysis. Using a combination of RNA-seq, ATAC-seq and H3K27ac ChIP-seq, the NC transcriptome and epigenomic landscape were characterized across four embryonic stages key to cNC development within the context of the foxd3-DNA binding landscape (Lukoseviciute et al., 2018). Foxd3 was shown to prime NC gene expression in early pre-migratory cNC by binding to its target enhancers. Conversely, later in cNC development, it represses active enhancers associated with mesenchymal/neuronal genes in line with previous *in vitro* data (Krishnakumar et al., 2016; Respuela et al., 2016). In short, using multiomics to characterize the *foxd3*-GRN *in vivo* across cNC developmental stages revealed the transition between gene modules as *foxd3* shifted toward its canonical repressive activity after NC specification. This can be achieved by switching binding partners, a phenomenon that has been observed with another NC pioneer factor TFAP2A as it imposes its function in NC induction and specification modules by dimerising with TFAP2C or TFAP2B, respectively (Rothstein and Simoes-Costa, 2020).

### 3.2. Vertebrate Evolution

From an evolutionary perspective, NC enhancers are a distinct group of components within the cNC-GRN that are molded under evolutionary pressure leading to species divergence of craniofacial structures (Prescott et al., 2015). Due to their heavy contribution to the patterning of vertebrate craniofacial structures (reviewed in Santagati and Rijli, 2003), the cNC is of particular interest as a key contributor to the evolution of jawed vertebrates (Cerny et al., 2010). This is supported by candidate-based approach evidence in lamprey, a basal vertebrate, highlighting functional interactions between main components of the GRN underlying NC ontogeny (Sauka-Spengler et al., 2007; Nikitina et al., 2008). Genome-wide studies in the lamprey were initially inhibited due to programmed large-scale genome loss during embryonic development (Smith et al., 2009), hampering acquisition of meaningful *omics* information despite the clear benefit for a more genome-wide approach as demonstrated in the basal chordate amphioxus (Yu et al., 2008). Publication of the lamprey germline genome (Smith et al., 2018) was a significant step forward in this regard and presented renewed opportunities to dissect the lamprey cNC-GRN using multiomics. By examining the transcriptional profiles of dorsal neural tube tissue containing the cNC, modules that were both previously known in other vertebrates and unique to the lamprey were identified (Hockman et al., 2019). Concurrently, another study highlighted the resemblance of lamprey cNC to amniote trunk NC (Martik et al., 2019). Nevertheless, by additionally analyzing ATAC-seq profiles in dorsal neural tube tissue, novel *cis*-regulatory elements for two lamprey NC-GRN players—*Tfap2B* and *SoxE1* were discovered. Strikingly, the lamprey SoxE1 enhancer was shown to be active in cNC-derived craniofacial features following integration into the zebrafish genome as well as in the amniote model, highlighting the potential for deep conservation of TF/enhancer interaction of NC-GRN enhancers (Hockman et al., 2019).

## 4. Future Directions

### 4.1. Non-coding RNAs Provide an Additional Facet to the cNC-GRN

RNAs derived from enhancers (enhancer RNAs or eRNAs) emerged following a study describing developmentally-regulated enhancers in mouse cortical neurons (Kim et al., 2010). Further studies demonstrated the sensitivity of eRNA induction as a hallmark of cellular response to biological stimuli (Wang et al., 2011a; Lam et al., 2013; Li et al., 2013), and suggested that eRNA transcription can be correlated to regulation of chromatin looping (Melo et al., 2013; Hsieh et al., 2014). While these *in vitro* studies painted an early picture of eRNA expression and their potential function in regulating enhancer-mediated gene expression, mechanistic details surrounding these observations remained elusive. Later studies attempted to address this conundrum by focusing on eRNA crosstalk with the chromatin landscape (Kaikkonen et al., 2013; Mousavi et al., 2013), eRNA potential function as molecular partners during gene regulation (Schaukowitch et al., 2014; Sigova et al., 2015), as well as attempted to distinguish between functionality of eRNA transcription or their RNA transcripts (Paralkar et al., 2016). Several recent studies have further shed light on eRNA transcription as a global indicator of activated gene expression programmes. Interrogation of the nuclear transcriptome of migrating cNC cells in zebrafish embryos detected bidirectional transcription at a global scale. This “feature” enabled clustering of putative enhancers that were also functionally associated with known NC genes (Trinh et al., 2017), in line with a previous report that suggested eRNA profiles were more indicative of enhancer activity compared to H3K27Ac ChIP-seq profiles (Zhu et al., 2013). A study by the FANTOM consortium further showed that genome-wide eRNA transcription appeared to be temporally regulated, often preceding transcription of associated protein-coding genes (Arner et al., 2015). In short, regardless of the biological function of eRNAs during development, their phenomenon in itself is able to highlight active regions of the non-coding genome. Therefore, characterization of eRNA transcriptomes has strong potential to inform on genome regulation mechanisms underlying the cNC-GRN.

Another class of under-explored non-coding RNA in development are long non-coding RNAs (lncRNAs). Landmark findings describing lncRNAs in the HOX locus, HOTAIR and HOTTIP, served as important case studies of modern lncRNA biology (Rinn et al., 2007; Tsai et al., 2010; Wang et al., 2011b). HOTAIR silenced gene expression at promoter regions of the HOXD locus by interacting with the chromatin remodeler Polycomb Repressive Complex 2 (PRC2) to facilitate H3K4 demethylation. Similar to the molecular mechanism of HOTAIR, HOTTIP from the HOXA locus was shown to recruit WDR5/MLL complexes and drive H3K4 trimethylation to activate gene transcription. Hence, coupling between lncRNA function and epigenetic regulation serves as a useful framework to address the roles of lncRNAs in GRNs underlying developmental programmes. It is also important to note that lncRNAs are not a completely novel discovery, as their presence at loci of imprinted genes were reported in the past. More recently, mechanisms of these “classical” lncRNAs were studied in detail. The “lncRNA-mediated chromatin regulation” model presented by HOTAIR and HOTTIP were echoed in studies involving *Airn, H19*, and *Xist* (Engreitz et al., 2013; Monnier et al., 2013; Santoro et al., 2013). Last but not least, RNA species from another class of non-coding RNA—microRNAs (miRNAs)—were also found to play roles in NC development with several candidates identified thus far (reviewed in Weiner, 2018). LncRNA and miRNA activities are not mutually exclusive and crosstalk between the two classes have been documented (Zheng et al., 2014; Tan et al., 2015). Altogether, the contribution of the non-coding genome serves as another exciting facet to development and evolution of the cNC-GRN—an uncharted territory ripe for exploration in the multiomics era.

### 4.2. Compartmentalizing NC Molecular Identity

**Figure 2:**
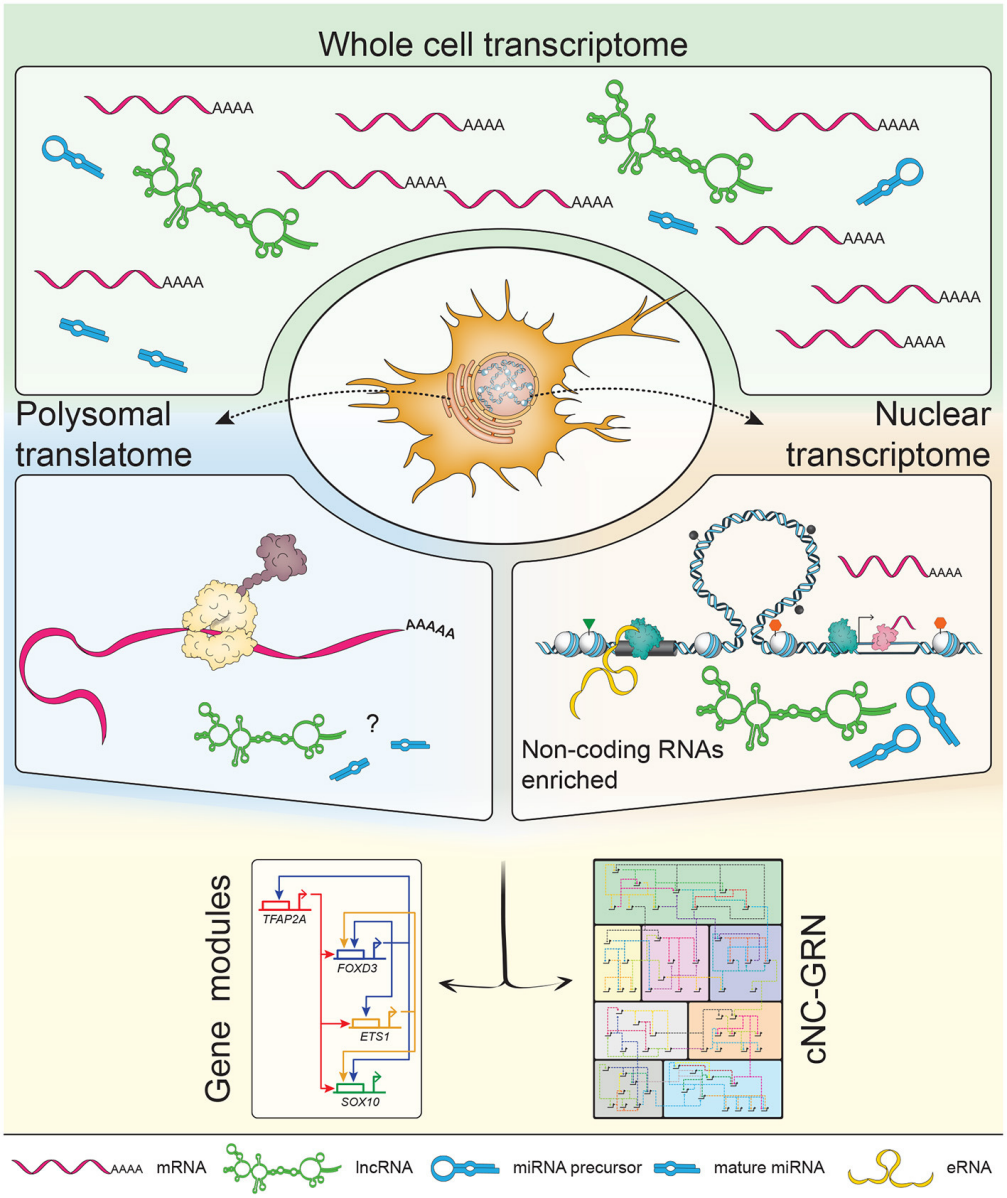
Subcellular profiling increases resolution of the non-coding landscape. The transcriptome consists of a mixed population of protein-coding and non-coding RNAs, including but not limited to enhancer RNAs (eRNAs), long non-coding RNAs (lncRNAs) and microRNAs (miRNAs). Previous transcriptomic studies on populations of neural crest (NC) cells focused on polyadenylated mRNAs constituting mostly of protein-coding mRNAs. NC-specific subcellular profiling achieved by *in vivo* biotinylation of nuclei and ribosomes (i.e., polysomes) enables enrichment of RNA species subtypes already present in the whole cell transcriptome. The nuclear transcriptome provided higher definition of non-coding RNAs while the polysomal translatome minimized the “noise” of non-coding RNAs to inform on proteins being made (suggestive of dominant biological processes occurring at that stage). In depth exploration of non-coding RNAs' putative roles within the context of the cNC-GRN is currently underexplored but well-suited to the advantages provided by multiomics.

Genome-wide profiling of polyadenylated transcripts from whole cell lysates provides a comprehensive snapshot of NC-GRN players being expressed at developmental stages-of-interest. Profiling polyadenylated transcripts alone, however, directly excludes non-polyadenylated RNAs enriched in the nucleus which form a large proportion of non-coding RNAs from intergenic regions (Carninci et al., 2005). This limitation can be addressed by subcellular profiling and rRNA-depletion during the construction of sequencing libraries. Isolating polysomes using recently-developed TRAP method (Heiman et al., 2014) and their associated mRNAs in the zebrafish migratory NC at 16–18 ss informed us of both known and novel NC markers forming the translatome at this developmental window (Chong, 2017). Enrichment of *elavl3* suggested that at least a subset of these cells (i.e., actively migrating cNC and premigratory trunk NC) were actively differentiating into their neuronal derivatives. On the other hand, by isolating nuclei transcriptomes at the same developmental stage, we demonstrated that functional annotation of transcribed enhancers (eRNAs) and promoters reflected the molecular signature of migratory NC cells and derivatives (Trinh et al., 2017); however the corresponding genes were not being translated as yet. Thus, similar to chromatin accessibility profiles which pre-defined cellular identities of cranial and vagal NC prior to associated gene expression (Ling and Sauka-Spengler, 2019; Williams et al., 2019), eRNA profiles also preceded coding gene transcription, thus reflecting future steps in NC ontogeny. Taken together, the translatome data provides a clearer picture without the “noise” from cytoplasmic or nuclear transcripts and suggested that at a given time-point, specification and/or differentiation of neuronal derivatives seem to dominate over ectodermal, mesodermal, and neuroepithelial derivatives (depleted in the translatome). These findings also highlight the utility of technologies to genetically attain subcellular resolution using *in vivo* biotinylation (de Boer et al., 2003; Deal and Henikoff, 2010; Trinh et al., 2018) with sufficient clarity to elucidate the role of non-coding RNAs in the cNC-GRN (Figure 2).

The contrast between the two pictures painted from nuclei and polysomes of NC cells at the same developmental stage raised important questions relating to our interpretation of how development proceeds at the cellular level. Traditionally, cellular identity has perhaps been defined over-simplistically via the expression of all protein markers in a GRN. We are now in the position to expand this definition by not only taking into account what *protein(s)* and where *within the organism* these players are involved, but also what *non-coding element(s)* are responsible for gene activation and where *within a cell* these new players are exerting their functions. Integrating this information is a next complex task on the agenda and is non-trivial given that NC cells transition from being a stem cell-like population to many subpopulations committed to different, not necessarily binary fates. It is therefore crucial to perform and integrate multiplex genetic lineage tracing analyses into this picture, interpret multiomics data at single cell and with subcellular resolution, as well as develop new, non-biased functional genomics integration tools based on artificial intelligence and deep learning approaches.

## 5. Discussion

Embryology has progressed in leaps and bounds leading to the modern incarnation of developmental biology as we know it today. From embryological techniques to advances in genome biology, our understanding of animal development has reached impressive heights. Here, at the forefront of modern developmental genetics and genomics, we propose using a combination of “traditional” and “modern” methods to deepen our understanding of genetic programmes underlying cNC development encoded within the genome.

The cNC is a multipotent population of cells key to vertebrate evolution. It is a versatile system for interrogation, as the genetic machinery underlying its biology reiterates throughout development and disease. This well-oiled system is also sensitive to fine-tuned regulation; disrupt a cog and development fails to proceed normally leading to neurocristopathies that account for roughly 1/3 of all birth defects. In order to discover ways to prevent or treat them, we first need to fully understand what the baseline scenarios are, at the level of genes within the context of a highly dynamic genome.

Tackling the non-coding genome has also uncovered non-coding RNA molecules that form part of the genetic regulation underlying cellular function. Previous work by many research groups has highlighted lncRNAs as molecular scaffolds that shuttle proteins to their target regions to regulate gene expression. eRNAs not only serve as “indicators” of when gene transcription onsets, but also have been proposed to facilitate chromosome-looping between enhancers and the promoters they regulate. Coupled with advances in gene editing including CRISPR/Cas, we are now in the position to design experiments with flexibility, efficiency and precision, from genome-wide screens of non-coding elements (Liu et al., 2016; Sanjana et al., 2016; Zhu et al., 2016) to *in vivo* decommissioning of NC enhancers for functional investigation (Williams et al., 2019).

In conclusion, we hope to not only propose fresh perspectives and potential avenues of investigation into the cNC-GRN but also challenge the reader to revisit how we study developmental biology as a whole. We are now ushering a new generation of scientists willing to embrace the exponential growth of molecular and computational tools at their disposal—the future is bright.

## Data Availability Statement

The original contributions presented in the study are included in the article/supplementary material, further inquiries can be directed to the corresponding author/s.

## Author Contributions

VC-M and TS-S contributed to the writing and editing of the manuscript. Both authors contributed to the article and approved the submitted version.

## Conflict of Interest

The authors declare that the research was conducted in the absence of any commercial or financial relationships that could be construed as a potential conflict of interest.
